# Nanomotors Sense Local Physicochemical Heterogeneities in Tumor Microenvironments**

**DOI:** 10.1002/anie.202008681

**Published:** 2020-10-25

**Authors:** Debayan Dasgupta, Dharma Pally, Deepak K. Saini, Ramray Bhat, Ambarish Ghosh

**Affiliations:** ^1^ Centre for Nano Science and Engineering Indian Institute of Science Bangalore 560012 India; ^2^ Department of Molecular Reproduction, Development and Genetics Indian Institute of Science Bangalore 560012 India; ^3^ Centre for Biosystems Science and Engineering, IISc Bangalore 560012 India; ^4^ Department of Physics Indian Institute of Science Bangalore 560012 India

**Keywords:** adhesion, cancer, extracellular matrix, sialic acid, tumorigenesis

## Abstract

The invasion of cancer is brought about by continuous interaction of malignant cells with their surrounding tissue microenvironment. Investigating the remodeling of local extracellular matrix (ECM) by invading cells can thus provide fundamental insights into the dynamics of cancer progression. In this paper, we use an active untethered nanomechanical tool, realized as magnetically driven nanomotors, to locally probe a 3D tissue culture environment. We observed that nanomotors preferentially adhere to the cancer‐proximal ECM and magnitude of the adhesive force increased with cell lines of higher metastatic ability. We experimentally confirmed that sialic acid linkage specific to cancer‐secreted ECM makes it differently charged, which causes this adhesion. In an assay consisting of both cancerous and non‐cancerous epithelia, that mimics the in vivo histopathological milieu of a malignant breast tumor, we find that nanomotors preferentially decorate the region around the cancer cells.

## Graphical abstract

Within a reconstructed tumor‐like 3D microenvironment, magnetically driven swarms of nanomotors selectively adhere to the extracellular matrix (ECM) surrounding breast cancer cells in a charge‐dependent manner. The ECM proximal to untransformed breast epithelia does not show adhesion. This principle is relevant for theranostic modalities seeking to localize and target cancer micro‐niches surrounded by healthy tissue.

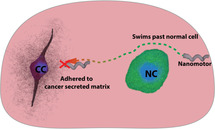

## Introduction

Cancer cells invade into their surrounding healthy tissues by degrading their surrounding extracellular matrix (ECM) and secreting fresh ECM.[^1^, ^2^] The altered matrix microenvironment in turn deregulates the signaling within cancer cells, potentiating their invasion.[^3^, ^4^] A strong interdependency exists between the ECM and cancer cells: mechanical properties of the ECM dysregulates signaling within cancerous cells, resulting in aberrant expression of matrix and matricellular proteins, which in turn can alter the chemical and physical composition of the ECM. Therefore, a quantitative understanding of physicochemical changes in cancer‐remodeled ECM is of great importance in the investigation of the mechanisms of cancer invasion and metastasis and understanding the formation and spread of cancer.^[5]^ Such measurements are not trivial, considering the composition of the cancer ECM is extremely heterogenous, arising due to three‐dimensional localization of the fibrillar network of biopolymers like collagen, along with various non‐fibrillar matrix proteins, proteoglycans, secreted factors and enzymes.[^6^, ^7^] As an alternative to traditional studies conducted on planar (two dimensional (2D)) cell cultures, current investigations have moved on to cells within three dimensional (3D) matrices, which mimic many of the physicochemical and mechanical properties of tumors in vivo.[^8^, ^9^, ^10^, ^11^] Assays based in 3D present a higher degree of complexity since the ECM stiffness, limited porosity and extensive polymer crosslinking observed in such environments typify the matrix microenvironment of tumors in vivo. Therefore, 3D cultures can provide a model platform for biophysical investigations as well as test emergent cancer therapeutic strategies.[^12^, ^13^]

Apart from various imaging techniques, such as scanning electron microscopy of scaffolds used for tissue engineering,^[14]^ optical coherence tomography (OCT) using gold nanorods,[^15^, ^16^] time‐lapse confocal reflection microscopy (TL‐CRM)^[17]^ and multiphoton imaging techniques,^[18]^ an experimental investigation of the local heterogeneities of 3D ECM environment has only been attempted with mechanical probes such as atomic force microscopy (AFM),[^19^, ^20^] or particle tracking microrheology (PTM).[^21^, ^22^] While AFM studies are limited to probing the exposed surfaces of cells and ECMs, PTM measures the rheological parameters of, and trace out local mechanical anisotropies in, the bulk matrix. However, measurements with untethered probes, such as used in PTM, are limited to a spatial range given by $\sqrt{Dt_{e}}$ , where D and $t_{e}$ refer to the diffusivity of the probe and duration of experiment, respectively. Also, the probe positions are randomized due to thermal fluctuations, which prohibits making measurements at observer‐specified locations.

Herein lies a major motivation of our study: can we drive the probes actively to measure the local heterogeneities of the ECM in well‐defined and specifically chosen locations at high speeds, and perform quantitative measurements of the local surroundings? As we show here, this can be realized with remotely controlled motile nanomotors moving in an ECM containing diverse epithelia, wherein the active probe can sense, map and even quantify cancer‐induced heterogeneities within the ECM.

In this paper, our technique was based on helical nanostructures containing ferromagnetic elements, injected in different regions of the recreated breast tumor microenvironment. The cells were embedded within a reconstituted basement membrane (rBM) matrix, which mimics the ECM that breast cancer epithelia interact with, degrade and replace with freshly synthesized collagenous ECM during invasion.^[23]^ The injected nanomotors (also referred to as nanorobots or nanopropellers in literature) were subjected to rotating magnetic fields, where the chiral shape caused the rotating nanomotors to move forward or backward, depending on the handedness of the helix and sense of rotation of the field. This method[^24^, ^25^, ^26^, ^27^] of remote manipulation of untethered nanomotors has been shown to be more efficient^[28]^ than conventional magnetic gradient pulling methods at small length scales. They also provide a promising route toward futuristic drug delivery vehicles, studies of cellular biophysics ^[29–33]^, microfluidic manipulation ^[34]^, sensing applications ^[35–39]^ and selective sorting of cancer cells.[^40^, ^41^] Indeed, these and similar nanostructures have been actively maneuvered in complex biological environments in the past;[^33^, ^38^, ^42^] however, we must stress that our findings are fundamentally different from all previous experiments. While the primary motivation in the past was to demonstrate maneuverability of the nanomotors in important biological environments, here, we use variations of maneuverability as a tool to quantify the changes in physical properties of the ECM near cancer cells. These differences between cancer cells and surrounding normal cells make important contributions to our knowledge of the heterogeneity of tumor microenvironment.

## Results

**Figure 1:**
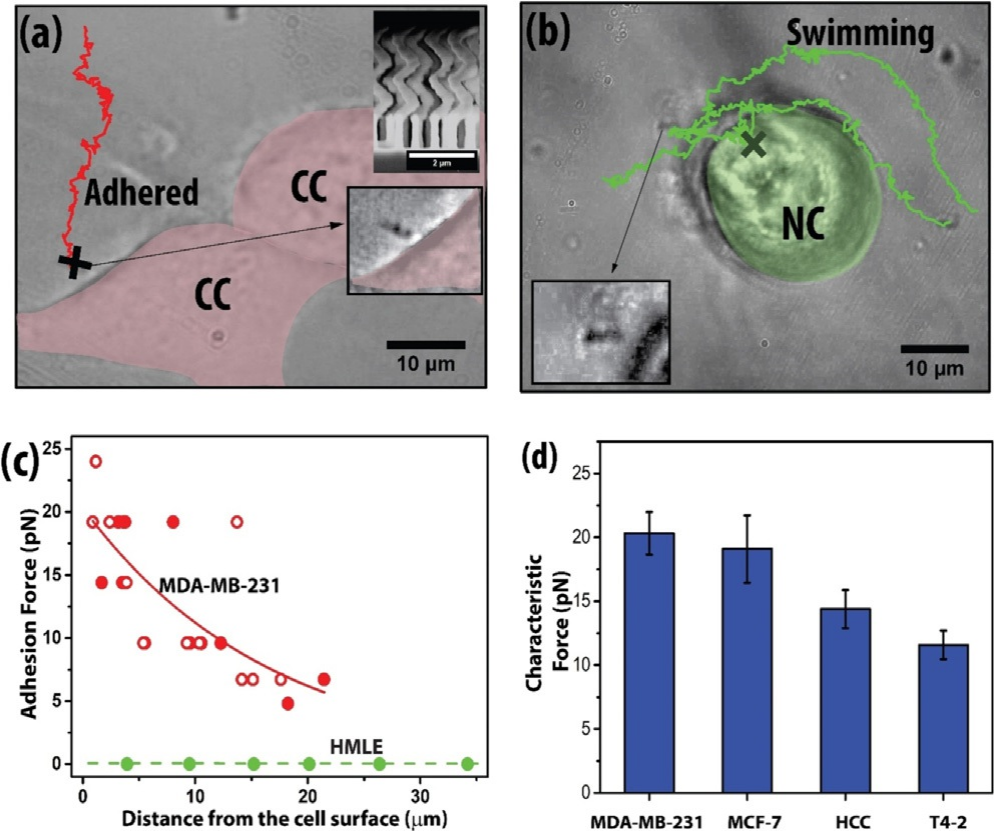
Nanomotors moving in ECM containing cancer and normal cells. a) Nanomotors driven in ECM getting adhered to the ECM near a cancer cell (denoted as CC), where CC has been pseudo‐colored in red to show the boundary. Inset showing scanning electron micrograph (scale bar is 2 μm), cross marking the location where the nanomotor get adhered. b) Nanomotors driven near a normal (non‐transformed, NC) cell without any adhesion. It eventually adheres directly on the surface of the normal cell, again marked by a cross. NC has been pseudo‐colored in green to show the cell boundary. c) Measurement of the adhesive force experienced by the nanomotor as a function of distance for CCs (MDA‐MB‐231, see movie M1). The solid and open red circles correspond to different techniques of force estimation, as described in the main text. Also shown (green dots and green dashed line) are measurements for the NCs (HMLE, see movie M2). d) Characteristic adhesive force for various cancer cell lines shown as bar graphs (see materials and methods for details on the statistics). One‐way ANOVA shows significant difference between the means of the four groups (*p*<0.0001).

A collection of nanomotors were injected into the tumor‐like environment, constituted as a 3D matrix scaffold of rBM, within which metastatic breast cancer cells MDA‐MB‐231 (which we call CC) or non‐cancerous breast epithelia HMLE (which we call NC) were cultured (see Materials and Methods for more details on fabrication and 3D culture). A brief description of the experimental setup, nanomotor fabrication and motion in rBM can be found in the supplementary information (SI: Section S1).The helical structures (see Figure 1 a inset) were made of silica with embedded iron particles, and were fabricated by Glancing Angle Deposition.^[24]^ These were confirmed to be biocompatible[^34^, ^43^] (see SI: Section S2). Cell‐embedded matrix scaffolds along with nanomotors was placed inside the Helmholtz coil and observed through a fluorescence microscope. The nanomotors were moved unidirectionally up to a millimeter for 30 minutes under a rotating magnetic field of constant strength and frequency. As shown in Figure 1 a, we observed that almost all of the nanomotors approaching CC adhered to the ECM in its vicinity (≈10–40 μm) (also see Movie M1 and SI:Figure S2 for additional images of adhered nanomotors imaged at higher magnification). On the other hand, a nanomotor would be able to approach very close to the NC and subsequently adhere on the NC surface or swim past the NC‐proximal ECM as shown in Figure 1 b (see Movie M2). The adhesion to surface of NC could be explained by the presence of sugar‐containing surface brushes, as reported previously.^[44]^ One must note an important difference: the NC were embedded in rBM in the experiments described in this manuscript, while previous measurements were carried out in monolayer cell cultures. The most important observation is the adhesive nature of the CC‐proximal ECM, which was not observed in NC‐proximal ECM. As far as we know, this difference has never been observed.

Next, we made quantitative measurements of the force of adhesion as a function of the distance x of the adhered nanomotor from the cell surface. The field B at which the nanomotor got adhered to the ECM could be related to the maximum available magnitude of the magnetic torque $\left|\vec{\tau }\right|$ :

(1) $$\left|\vec{\tau }\right|=\vec{m}\times \vec{B}=\frac{\Omega_{1}\eta f_{g}}{\sin\left(\theta_{m}\right)},$$

which in turn can be used to calculate the effective magnitude of the adhesive force, given by:

(2) $$F=\frac{2\tau }{t}=\frac{2\Omega_{1}\eta f_{g}}{t\;sin\left(\theta_{m}\right)},$$

where, in the above two expressions, force is given by F , m is the magnetic moment of the nanomotor and t is the filament diameter of the helix, $\Omega_{1}$ is a characteristic cutoff frequency (see SI: Section S3), η is the viscosity of the fluid, $f_{g}$ is a geometrical factor related to the drag coefficient of a rod rotating about the short axis, and $\theta_{m}$ is the angle between the direction of magnetization and the short axis of the helically shaped nanomotor. The strength of applied field lay between 50 and 250 Gauss. The method to estimate m has been described in previous papers[^35^, ^45^] from our group and also discussed in the materials and methods section. The adhesive force, shown as solid red circles in Figure 1 c, was estimated from the thrust exerted by the nanomotor that was just enough to drive it to the point in the ECM where it got adhered. A higher field would be necessary to overcome the adhesion and move further near the cell membrane.

For a few nanomotors near CC, it was indeed possible to get the motor detached from the ECM by just increasing the field strength by another 50 or 100 Gauss. The corresponding measurements are intrinsically self‐referenced in nature, since the same nanomotor shows attachment to the ECM and subsequent detachment under a higher field. The estimates of adhesion force for nanomotors, which could be detached are represented by the red open circles in Figure 1 c. However, detachment from ECM was not always possible, as the highest field strength of 250 Gauss available from our setup was not always enough to generate the torque needed to move an adhered nanomotor.

The maximum force of adhesion provided by the NC, where adhesion was not observed, was estimated by calculating the drag overcome by the nanomotors while swimming in the matrix, as shown in Figure 1 c as green dot symbols. We estimated the force F as:^[46]^

(3) $$F=\frac{4\eta \pi aV}{\left[\ln\left(\frac{2a}{b}\right)-\;\frac{1}{2}\right]},$$

Where the shape of the helix is approximated as an ellipsoid with a and b being the dimensions of the semi‐major and semi‐minor axes, respectively, η the viscosity of the surrounding medium, and V is the speed of the nanomotor.

To quantify the adhesion further, we fit an exponential function:

(4) $$F=F_{0}exp\left(\frac{-x}{l_{p}}\right),$$

where x is the distance of the nanomotor from the cell surface. The fitting has two parameters which represent characteristic force ($F_{0}$ ) and characteristic length scale ($l_{p}$ ) for different cell lines. We used four different CC lines with variable extents of invasiveness and metastatic ability: MDA‐MB‐231, a triple negative cancer cell line that shows an invasive “stellate”‐like growth phenotype, when cultured on rBM; MCF‐7, HCC70 and T4−2 represent three breast cancer cell lines that show a milder “mass”‐like morphologies on rBM matrices[^47^, ^48^] (See SI: Table S1 for a description of the cell lines used in this study. Also see SI: Figure S4 for adhesion force calculation in other cell lines). Along with this, two different NC lines HMLE and S1 were used. In Figure 1 d, the fitting of the exponential function was done on data obtained from three separate cell‐culture dishes for each cell lines. In total, 25 cell‐nanomotor events were obtained from observing 17 different MDA‐MB‐231 cells, 15 cell‐nanomotor events obtained from observing 13 different MCF‐7 cells, 15 cell‐nanomotor events from observing 13 different T‐42 cells and 17 cell‐nanomotor events from 17 different HCC cells. The variation of $F_{0}$ across different cell lines, as shown in Figure 1 d, suggests the adhesive force to be greater for cells with higher metastatic potential. The characteristic distance $l_{p}$ was found to be ≈18 μm across all the four cell lines studied in our experiments.

We must stress the adhesion studies presented here are fundamentally different from previous reports of silica AFM probes adhering to surfaces of cancer and normal cells differently. These studies investigated differences in surface adhesion on the surface between cancer and normal cells.^[44]^ On the other hand, our findings pertain to modification of the surrounding ECM caused by cancer.

PFO‐coated nanomotors do not adhere to the ECM despite being near the CC, unlike uncoated motors, which adhere to the ECM nearby.

**Figure 2:**
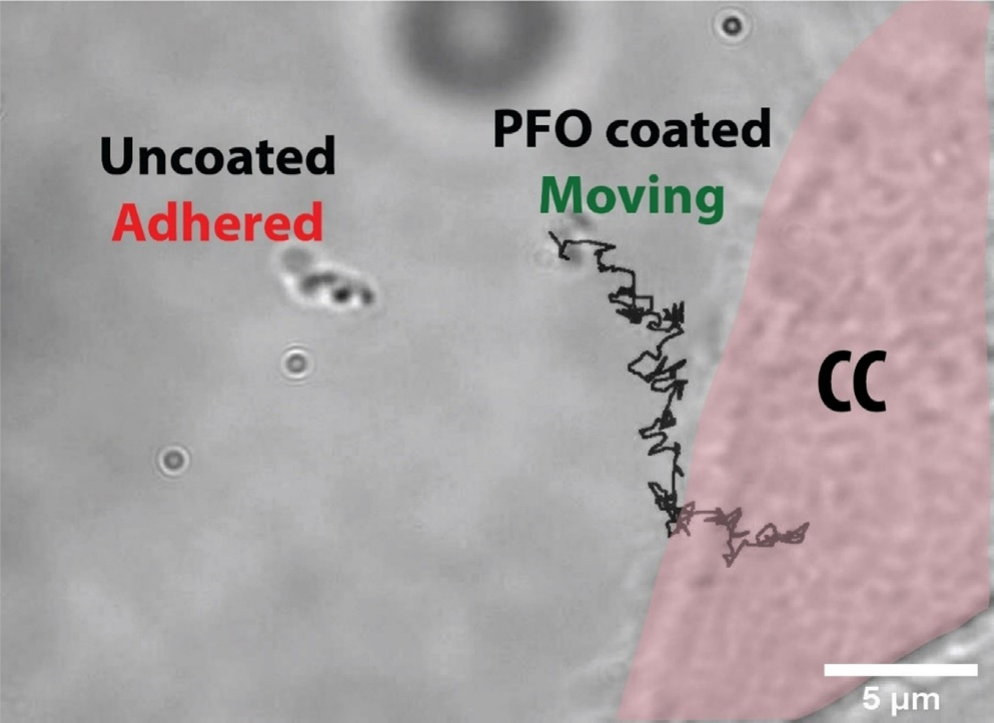
Effect of 1*H*, 2*H*, 2*H*‐Perfluorooctyltriethoxysilane (PFO) coating on nanomotors.

The data presented so far clearly indicates the presence of an adhesive factor near CC, which is absent in the ECM surrounding NC. Considering cancer microenvironment is mostly comprised of charged proteins, which are known to regulate steps of carcinogenesis,^[49]^ we hypothesized that a charge‐based mechanism could explain this differential adhesive property. To confirm this hypothesis, we coated the nanomotor surface with 1*H*, 1*H*, 2*H*, 2*H*‐Perfluorooctyltriethoxysilane (PFO), which shields the surface of the nanomotor from charged environments. The surface‐coating with PFO was confirmed using FTIR (see SI: Figure S5). We observed that the adhesion of PFO‐coated nanomotors to ECM in the vicinity of the CC was substantially reduced when compared with uncoated controls. For example, as shown in Figure 2, where both PFO‐coated and uncoated nanomotors were simultaneously driven at 150 Gauss magnetic field rotating at 3 Hz (see movie M3), we observed the uncoated nanomotors to stick in the neighborhood of the CC away from the surface, while the PFO‐coated nanomotors remained unadhered even very close to the CC, thus confirming our hypothesis. This experiment was repeated on 3 different cell culture dishes where the same behavior was observed. Some more examples of PFO coated nanomotors approaching CC without adhesion can be found in the SI (See SI: Figure S6)

**Figure 3:**
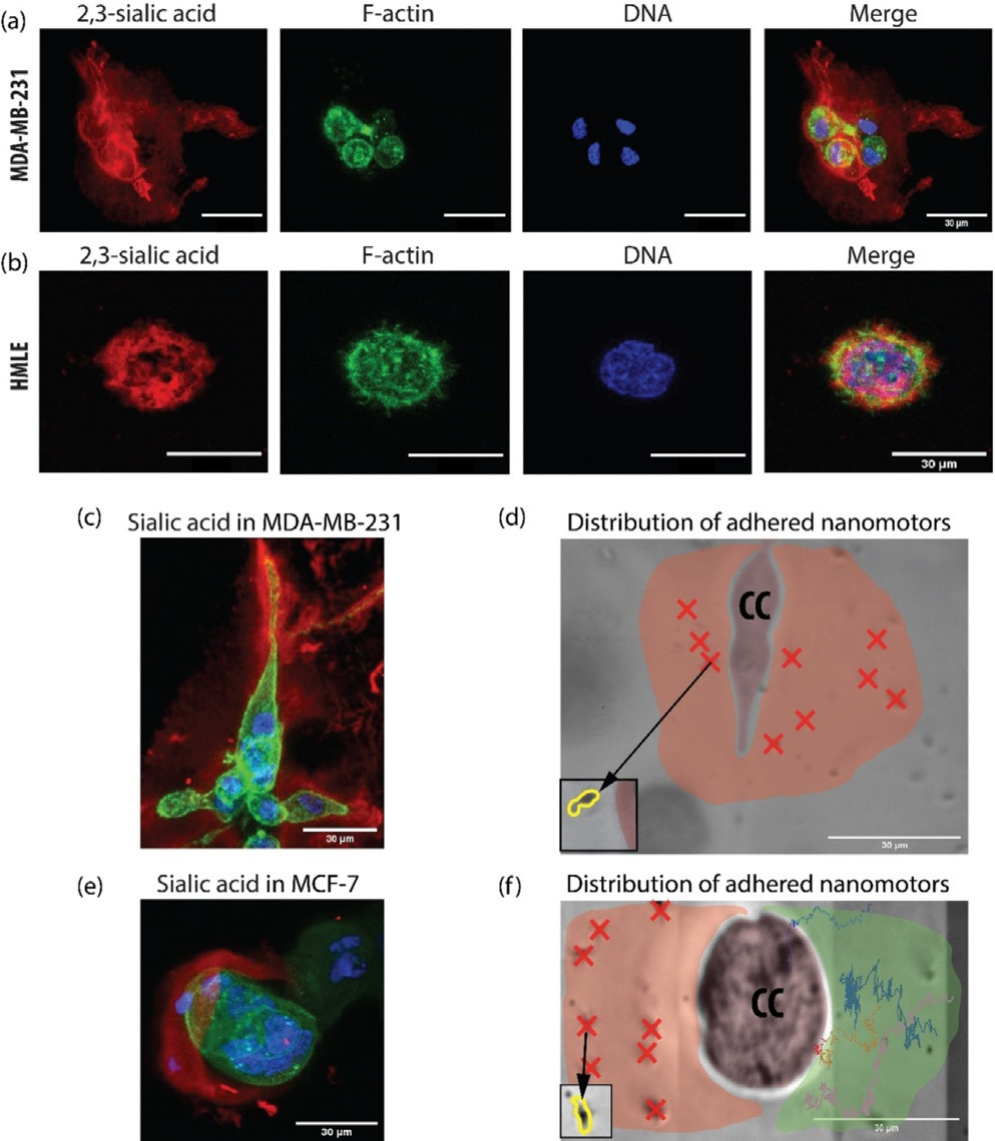
Role of sialylation in the preferential adhesion of the nanomotors near cancer cells. a) Confocal micrographs showing the distinct presence of α2,3‐linked sialic acid (red) in the ECM surrounding MDA‐MB‐231(CC) cells. The F‐actin (green) and DNA (blue) are stained to mark the cortical cytoplasm and nucleus, respectively (similar staining was observed in three independent biological repeats). b) In comparison with (a), only cell surface localization of α2,3‐linked sialic acid is evident in HMLE cells (NC) The sialyation was not evident in the proximal ECM. A 3D view of these projections is available in SI: Figure S8. c) Fluorescence image of the same confirms isotropic distribution of sialylated ECM. d) Representative brightfield image of MDA‐MB‐231 cell showing an isotropic spatial distribution of multiple adhered nanomotors. The region where nanomotors were found to adhere to ECM has been shaded in red. e) Fluorescence image in the right panel confirms the anisotropic distribution of sialylated ECM in MCF‐7 cells. f) Representative image of MCF‐7 observed under a brightfield microscope with nanomotors propelling towards the cell. The left region of the cell shaded in red has many motors adhered to the ECM, denoted by red crosses. The trajectories of nanomotors approaching from the right are plotted in the region shaded green. No adhesion was observed in the area shaded in green. All scale bars represent 30 μm.

While previous work has been done to study the interaction between 3D cell culture‐nanomotor while the motors penetrated the cells^[50]^ or 3D spheroids using chemically powered nanomotors[^51^, ^52^, ^53^, ^54^, ^55^, ^56^] and acoustically propelled microdrills,[^57^, ^58^] here we look at the interaction of the nanomotors with the cancer modified microenvironment. We consider the molecular mechanism by which the nanomotors adhere to the cancer microenvironment is through electrostatic, that is, charge‐based interactions. The cancer microenvironment is abundant in sugar‐conjugated molecules, which are present both on cell surfaces (and form their brushes) and in the ECM. Of these sugars, sialic acids are among the most well‐studied molecules to be aberrantly expressed in cancer and regulate disease progression.^[49]^ Given its additional property of imparting negative charge to its conjugates, we hypothesized that sialic acids may render the surrounding ECM highly negatively charged. Therefore, we used lectin fluorescence cytochemistry to assay for the spatial localization of sialic acids in our 3D cultures. TRITC‐conjugated MAA (*Maackia amurensis* Agglutinin) was used to detect α2,3‐linked sialic acids (red) whereas Phalloidin‐Alexa 488 was used to visualize cortical F‐actin (green) which is present at the cytoplasmic boundary as a surface marker as shown in Figure 3 a. It was observed that the spatial extent of α2,3‐linked sialic acid staining in CC extended to the surrounding ECM and was similar to the length scale (about 40 μm from the cell surface) at which nanomotors are shown to encounter adhesion around cancer cells (see SI: Figure S7). This distribution of sialylated proteins was limited to the cell surface and not observed in the ECM surrounding NC as shown in Figure 3 b. This suggests that CC‐secreted negatively charged α2,3‐linked sialoconjugated ECM may be the primary cause of nanomotor adhesion.

A striking observation was made with respect to the distribution of sialylated proteins in the ECM surrounding the cancer cells: MDA‐MB‐231 and MCF‐7. While the former, shown in Figure 3 c, revealed an isotropic spatial distribution of α2,3‐linked sialic acids in its surrounding ECM, we observed presence of this sugar linkage mostly confined to one side of MCF‐7, implying anisotropic modification of the proximal ECM by MCF‐7, as shown in Figure 3 e. The same anisotropic behaviour was observed when many nanomotors were driven toward the cells from all directions. As shown in Figures 3 d and f, the nanomotors were found to be adhered isotropically to the ECM proximal to MDA‐MB‐231, but only on one side of MCF‐7. This further confirms the role played by the charged sialylated conjugates within CC‐proximal ECM in adhering the nanomotors.

**Figure 4:**
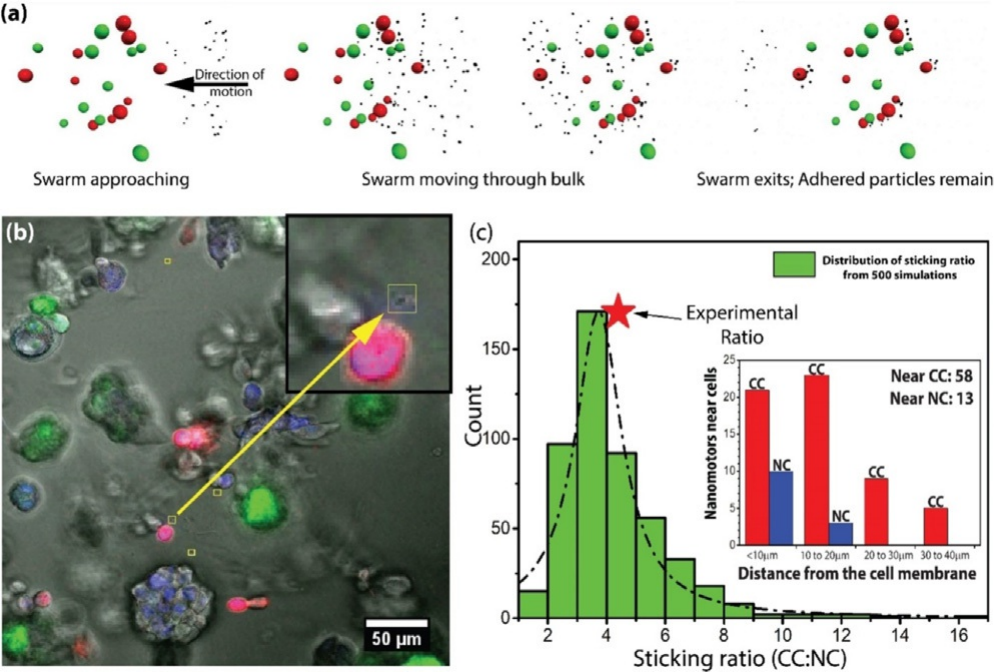
Experimental and simulation results in microenvironmental co‐cultures. a) Schematic showing strategy to localize nanomotors preferentially around the CCs in a co‐culture. Red, green spheres and black dots represent CCs, NCs and nanomotors, respectively. b) A single slice from a confocal stack showing nanomotors in the vicinity of CC (RFP MDA‐MB‐231). The NC are green (GFP HMLE). Inset shows a zoomed view of the nanomotor near the CC. c) Probability distribution of ratio of nanomotors adhered near CC: NC cells as obtained from 500 numerical simulations. Dashed line is guide to eye. Also shown, with red star is the experimental ratio obtained from sampling 376 individual cells (more details in the main text). Inset shows the spatial distribution of adhered nanomotors in co‐cultures where 71 cells were found to have nanomotors in their vicinity.

We extended our studies to microenvironmental co‐cultures, such as to test the possibility of using the differential adhesive property of the nanomotors as a strategy for targeting cancer cells. The idea behind this experiment is shown in the schematic in Figure 4 a. Assuming the 3D co‐culture contained both CCs and NCs, we expected that nanomotors swimming through the 3D culture would preferentially adhere to the CC‐secreted ECM, whereas a few may adhere to the surface on NC.

The co‐culture was constituted as a 3D matrix scaffold of rBM, within which metastatic breast cancer cells MDA‐MB‐231 (expressing red fluorescent protein (RFP)) and non‐cancerous breast epithelia HMLE (expressing green fluorescent protein, (GFP)) were cultured. The nanomotors were injected and subsequently driven within the matrix unidirectionally, for ≈30 minutes at 3 Hz rotating field of strength 50 Gauss. We examined a region more than 1000 μm away from the point of injection, which was significantly further than the distance covered by the motors through passive diffusion (see SI: Section S4). The confocal images (see movie M4) showed many nanomotors distributed in 3D. We examined representative examples taken from the stack of confocal images as shown in Figure 4 b, which showed most of the nanomotors to be present/adhered to ECM close to the cancer cells, in agreement with the results of the single culture experiment shown in Figure 1. We repeated this experiment on 5 different samples. In total 376 cells were sampled, far away from the point of injection of the motors, of which 203 cells were cancer cells and 173 were non‐cancerous cells. A total of 71 cells were found to have nanomotors adhered to the ECM in their vicinity, of which 58 were cancer cells and 13 were non‐cancerous cells as shown in the inset of Figure 4 c. For the adhesion close to non‐cancerous cells, nanomotors were found to be on the surface of those cells. These experiments allowed us to estimate the probability of a nanomotor to be adhered close to a cancer cell as approximately 4:1 higher compared to a normal cell, thus establishing a completely new strategy toward targeting cancer cells from within a heterogeneous cell population.

To validate these observations further, we performed a Monte Carlo based simulation of nanomotors moving through a region containing heterogeneities in local adhesive properties. We considered a volume of 350×350×250 μm^3^ in which 15 cancer and 15 normal cells were dispersed with a random spatial distribution. These numbers were chosen to mimic the experimental scenario. The swarm of nanomotors was assumed to be randomly dispersed initially, in a volume of similar magnitude kept 350 μm away. The swarm was moved toward the cells unidirectionally, with the rule that whenever the nanomotor collides with a normal cell (approximated to be a sphere of radius 15 μm), it would be irreversibly stuck. Similarly, the motors were assumed to adhere to the CC‐proximal ECM, up to 35 μm from the cell surface, as per the results shown in the inset of Figure 4 c. Please note the choice of 35 μm is also consistent with the measurements on MDA‐MB‐231 cells shown in Figure 1 c for similar experimental conditions. We show the simulated movie (see movie M5) for three different initial conditions, corresponding to different spatial configurations of the motors and the cluster of cells. These simulations were performed for 500 random distributions, each one providing the ratio of nanomotors stuck to CC‐proximal ECM compared to NC surfaces. The histogram of these ratios is shown in Figure 4 c. The distribution peaks at the CC:NC ratio of 3.5:1, in agreement with the experimental observations.

## Conclusion

In summary, we have demonstrated how investigation of maneuverability of the nanomotors can be used to spatially differentiate between cancerous and non‐cancerous cell niches that were engineered to coexist within a tissue‐like microenvironment. Our observations were consistent across four distinct breast cancer cell lines (and 2 non‐cancerous breast epithelial lines), which suggests this observation may be applicable across malignant tumors of other organs as well. We find that the physical effects of invading cancer cells manifest up to a length of ≈40 μm from the cell surface within the ECM surrounding them. The adhesive force measurements provide a direct insight into how the presence of charged sialic acids within ECM secreted by cancer cells can be correlated to their metastatic potential.

Hypersialylation of tumor cells has for long been demonstrated in multiple cancers;[^59^, ^60^, ^61^, ^62^] however, these study the effects on cancer cell behavior by sialic acids that are conjugated to glycoproteins localized on cell surfaces. In this manuscript, we observe specific sialic acid linkage within the ECM secreted by cancer cells. The proteins that are secreted by cancer cells and constitute their surrounding ECM are collectively referred to as the cancer matrisome,[^2^, ^7^] which has been demonstrated to be chemically distinct from that of normal cells.^[63]^ Here we show for the first time, that the cancer matrisome shows distinct signatures of sialylation: the latter may render its mechanochemical properties distinct from that of untransformed cells.

Cancer cells have been shown to remodel their surrounding matrix microenvironments through multiple mechanisms: through secretion of metalloproteinases that degrade matrix proteins,^[64]^ expression of lysyl oxidases that crosslink collagen fibers,^[65]^ and synthesis of proteoglycans that can modulate the fibrillar topography.^[66]^ We believe that our observed length scale of ≈40 μm may represent the spatial transition between the synthesis (proximal to the cancer cells) and degradative (distal) remodeling processes. Therefore, our findings suggest a simple and yet elegant method of targeting cancer cell niche with enough specificity. We believe these findings will find use as targeting strategies in future in vivo applications, in quantification of cancer aggression and as biophysical probes to study the extracellular environment of cancer.

Movie List:

M1: Adhesion of nanomotor near a cancer cell (Cell Type: MDA‐MB‐231)

M2: No adhesion near normal cells (Cell Type: HMLE)

M3: PFO coated and uncoated nanomotors (Cell Type: MDA‐MB‐231)

M4: Confocal stacks of co‐culture in 3D matrix with nanomotor co‐localization (Cell Type: MDA‐MB‐231 and HMLE)

M5: Simulation of nanomotors moving through microenvironmental co‐culture

## Conflict of interest

The authors declare no conflict of interest.

## Supporting information

As a service to our authors and readers, this journal provides supporting information supplied by the authors. Such materials are peer reviewed and may be re‐organized for online delivery, but are not copy‐edited or typeset. Technical support issues arising from supporting information (other than missing files) should be addressed to the authors.

Supplementary

Supplementary

Supplementary

Supplementary

Supplementary

Supplementary
