# Supplementary material for: Nanomotors Sense Local Physicochemical Heterogeneities in Tumor Microenvironments
Source: Angew Chem Int Ed Engl. 2020 Oct 25;59(52):23690–6. doi: 10.1002/anie.202008681 (PMC7756332; doi:10.1002/anie.202008681)
Supplement: Supplementary file 6 — Supplementary [file ANIE-59-23690-s006.pdf]

Angewandte Chemie
Eine Zeitschrift der Gesellschaft Deutscher Chemiker

Supporting Information

Nanomotors Sense Local Physicochemical Heterogeneities in Tumor Microenvironments**

Debayan Dasgupta[+], Dharma Pally[+], Deepak K. Saini, Ramray Bhat, and Ambarish Ghosh**

anie_202008681_sm_miscellaneous_information.pdf
anie_202008681_sm_M1.mp4
anie_202008681_sm_M2.mp4
anie_202008681_sm_M3.mp4
anie_202008681_sm_M4.mp4
anie_202008681_sm_M5.mp4

Materials and methods

Fabrication of thin nanomotors:

To fabricate thin nanomotors, it was necessary to reduce the seed layer size while maintaining enough distance between subsequent seeds to allow shadowing during evaporation. This was achieved by using Langmuir Blodgett layers of 700 nm polystyrene beads. After monolayer formation, the beads were etched down to 500 nm by air plasma etching. The etched sample was further subjected to reactive ion etching to create 1 µm pillars on the silicon wafer (see SI: Figure S1(b)). The top of the 1 µm pillars was coated with a thin film of 18 nm silver which was subsequently annealed at 300^0 C for 15 minutes to form silver balls of diameter 200 nm. This was used as the seed layer for Glancing Angle Deposition (GLAD) of silica in which the magnetic material made of iron and cobalt powder mixed in 1:1 (w/w) ratio was integrated inline during the shadow growth. This ensured encapsulation of the magnetic material by silica, thus shielding it from the external environment and preventing etching and degradation under extreme conditions.

Statistics:

Data was analyzed using Origin 9.1, MATLAB 2015b and ImageJ. All data are presented as mean ± standard deviations (SDs). Comparison of counts between distinct groups was made by t-test of proportions.

Cell culture:

HMLE cells were a kind gift from Prof. Robert Weinberg, Harvard Medical School and Dr. Annapoorni Rangarajan, Indian Institute of Science. These cells were cultured in DMEM:F12 (1:1) (HiMedia, AT140) supplemented with 1% fetal bovine serum (Gibco, 10270), 0.5 µg/mL Hydrocortisone (Sigma, H0888), 10 µg/mL Insulin (Sigma, I6634) and 10 ng/mL human recombinant epidermal growth factor (HiMedia, TC228). MDA-MB-231 and HCC70 cells were grown in DMEM:F12 (1:1) along with 10% fetal bovine serum. MCF-7 cells were maintained in DMEM (HiMedia, AT007F) supplemented with 10% fetal bovine serum. S1 and T4-2 cells were cultures as described elsewhere [1]. All the cells were maintained in a humidified chamber at 37^0 C temperature and 5% carbon dioxide.

3D culture and experimental procedure:

In case of 3D monoculture, 5×10^4 cells were mixed in 50 µl of rBM (Corning, 354230) and allowed to solidify at 37^0 C temperature and 5% carbon dioxide in a humidified chamber. The concentration of rBM was ~9 mg/ml. For co-culture, 2.5×10^4 of HMLE constitutively expressing GFP and 2.5×10^4 MDA-MB-231 constitutively expressing RFP cells were mixed in 50 µl of rBM and allowed to solidify at 37^0 C temperature and 5% carbon dioxide in a humidified chamber. Cultures were grown in a defined medium [1] for 72 hours before injecting the nanomotors. An area of 0.5 mm^2 of a wafer containing nanomotors was

sonicated into a microcentrifuge tube containing 50 μl deionized water. A 10 μl solution containing 10^5 nanomotors suspended in deionized water was injected into the 3D matrix using a 26-gauge syringe. The sample was placed in a triaxial Helmholtz coil mounted on an optical microscope (Olympus IX71) and imaged through a 50x (or 100x) objective lens. The nanomotors were observed while the field was on and recorded using a CMOS camera. It was observed that at the site of injection of nanomotors many of them got adhered to the glass slide. The presence of local pockets of injected fluids has been reported in a previous paper from our group[2], where we showed that within a time scale of approximately 30 minutes, the system gains its structural uniformity. To be completely sure that the experiments are carried out in native rBM-cell environment, we drive the nanomotors for 30 minutes; which is at least 1000 μm away from the site of injection. We observed 0-5 nanomotors per cell at the region of experiment.

For confocal imaging, the nanomotors were actuated at 50 Gauss field rotating with a frequency of 3Hz for 30 minutes. The sample was subsequently fixed using sucrose solution after actuation. The experiments for measuring the adhesive force was generally done using 5 different field strengths (70, 100, 150, 200 and 250 Gauss) and the actuating frequency was kept either at 3Hz or 5Hz.

α2,3-linked sialic acid staining:

3×10^4 cells (HMLE, MCF-7 and MDA-MB-231) were seeded on top of solidified rBM in a 8-well chambered cover glass (Eppendorf, 0030742036). Cells were grown for 72 hours in defined medium[1] at 37^0 C temperature and 5% carbon dioxide in a humidified chamber . Cells were washed with PBS and treated with 18% and 30% sucrose in Phosphate-Buffered Saline (PBS) respectively for 30 minutes each at room temperature. Cells were fixed with 4% formaldehyde (Merck,1.94989.0521) for 20 minutes at room temperature and washed with PBS twice. Cells were stained with TRITC conjugated *Maackia amurensis* Lectin (MAA/MAL I) (1:200) (bioWORLD, 21510007-1) overnight at 4^0 C. After MAA staining, cells were washed with PBS thrice, 5 minutes each at room temperature and counter stained with 4,6-diamidino-2-phenylindole (DAPI) (Invitrogen, D1306) and Alexa Flour 488-conjugated Phalloidin (Invitrogen, A12379).

Laser scanning confocal microscopy:

All the fluorescence images were captured using either Zeiss LSM 880 or Leica TCS SP8 confocal microscope with system optimized Z intervals. DAPI, Alexa-488 and Alexa-568 dyes were excited with 405 nm diode, 488 nm Argon laser and 543 nm He-Ne lasers with appropriate filters and beam splitters. Co-culture images were acquired using a Plan-apochromat 40X oil immersion objective with no digital offset and digital gain. Lectin cytochemistry images were acquired using 20X Plan-apochromat objective with no digital gain and digital offset. 2D projections of 3D images were generated using maximum intensity projection algorithm. At least three random fields were imaged in each experiment. Laser

intensity and detector gain threshold were decided using a negative control for each experimental condition. Images were processed/analyzed using either Zen lite or Fiji software [3].

Estimation of the magnetic moment: The standard method of calculating the magnetic moment of a nanomotor has been described in previous literature in great details. A microfluidic chamber was made using a coverslip on a glass slide, in which nanomotor solution was placed. The chamber was placed under the microscope in a Helmholtz coil and subjected to 30 Gauss field while the frequency was varied from 1Hz to 10Hz. The precession angle of the nanomotor was recorded and a frequency vs precession angle curve was plotted. The frequency, where the nanomotor changed its precession angle from 90^0 (tumbling state) to a lower angle (precession state) was calculated by proper fit. This was done at regular intervals over the time period when the experiments were conducted. Initial magnetic moment was calculated to be $\sim 1.01 \times 10^{-16} Am^2$. However, since the same sample was used for all our experiments involving force calculations, the magnetic moment was found to have weakened to a value of $5 \times 10^{-17} Am^2$ after a period of 12 months.

Coating of PFO on nanomotors: To coat nanomotors with PFO the wafer containing nanomotors was placed in vacuum with about 20 µl of PFO and left overnight. This was enough to coat nanomotors with PFO as seen from the FTIR data (SI: Figure S5). We also confirmed the PFO coating on glass surface using similar coating protocol. The contact angle for PFO coated glass surface was found to be 145^0.

Supporting information

Section S1: Motion of helical nanomotors

Helical magnetic nanomotors can be maneuvered using a triaxial Helmholtz coil as shown in the following Figure S1(a). For our experiments, the triaxial coil was controlled by a LabVIEW program through a DAQ connected to 3 amplifiers. This setup was placed inside a fluorescent inverted microscope. An SEM of the Glancing Angle Deposition film grown on silicon pillar seed layer is shown in Figure S1(b). Figure S1(c) shown snapshots from a movie where the nanomotors was seen moving through the reconstituted Basement Membrane (rBM) material.

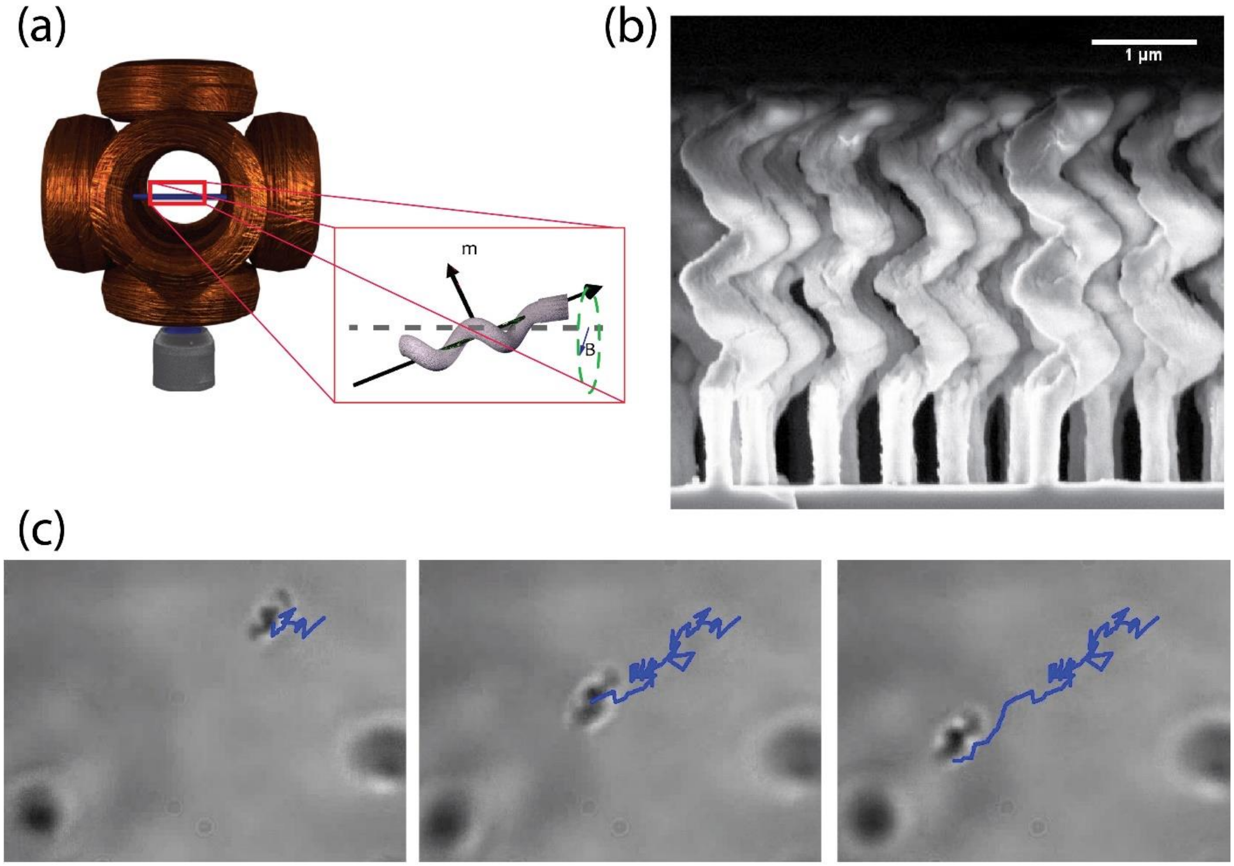

Figure S1:

(a) Schematic showing a triaxial Helmholtz coil and its placement inside a fluorescent microscope. (b) Electron microscope image of nanomotors fabricated using Glancing Angle Deposition. (c) Trajectory of a nanomotor moving through rBM.

Section S2: Cell Viability

5000 cells were seeded in each well of a 96 well plate. After overnight culture, cells were treated with 10^5 nanomotors for 24 hours to check if they have any effect on cell viability. After 24 hours, 100 μg/mL resazurin was added to each well and incubated for 2 hours. Fluorescence was measured at Ex 560 nm/ Em 590 nm as a read out for viability of cells. Fluorescence readings from treated cells was normalized to untreated cells. There was no change in viability of MDA-MB-231 cells upon treatment while HMLE cells show mild, insignificant decrease in viability.

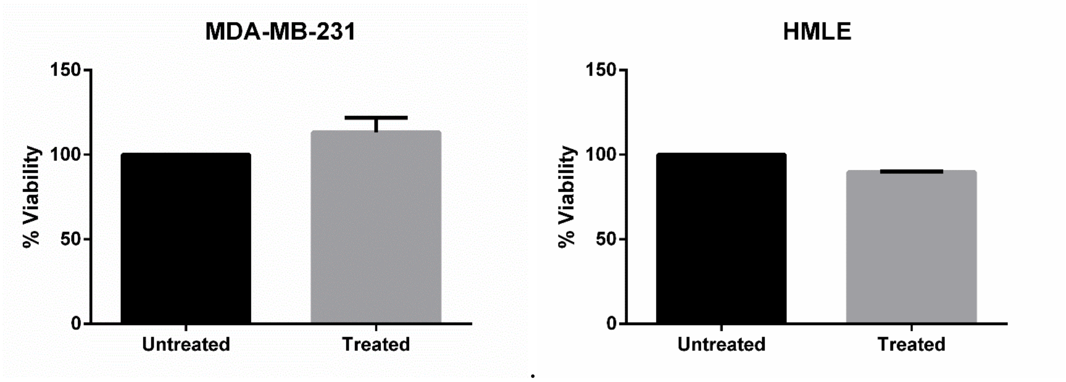

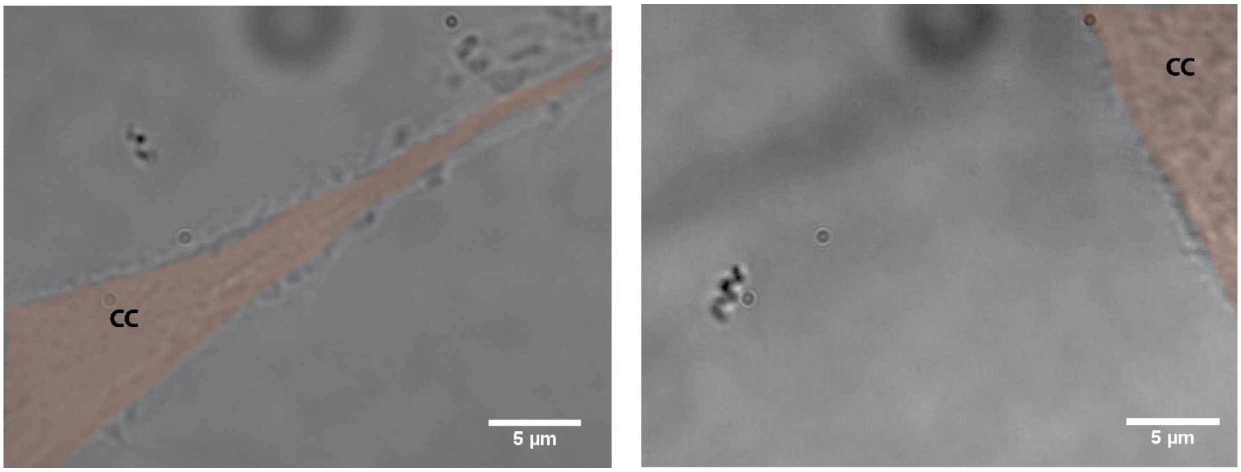

Figure S2: Examples of nanomotors adhered near CC

Section S3: Dynamics of a nanomotor

The torque used for calculating the force needed by the nanomotor to drive it to a point in the ECM where it gets adhered has been derived from two parameters m, B $(\tau = m \times B)$. m represents the magnetic moment vector of the nanomotor. $\theta_m$is the angle between the m vector and the short axis of the helix (see Figure S3). As mentioned in the main text there is a characteristic cutoff frequency Ω_1 which is needed to

measure the torque $\tau = m \times B = \frac{\Omega_1 \eta f_g}{\sin(\theta_m)}$. This was calculated by observing the dynamics of nanomotor for a range of frequency and measuring the precession angle at the imaging plane. The frequency where the nanomotor transitions from tumbling (Figure S3(a)) to precession (Figure S3(b)) was found by fitting the experimentally observed angle of rotation to $\sin^{-1}\left(\frac{\Omega_1}{\Omega_\mathrm{B}}\right)$ as shown in Figure S3(c).

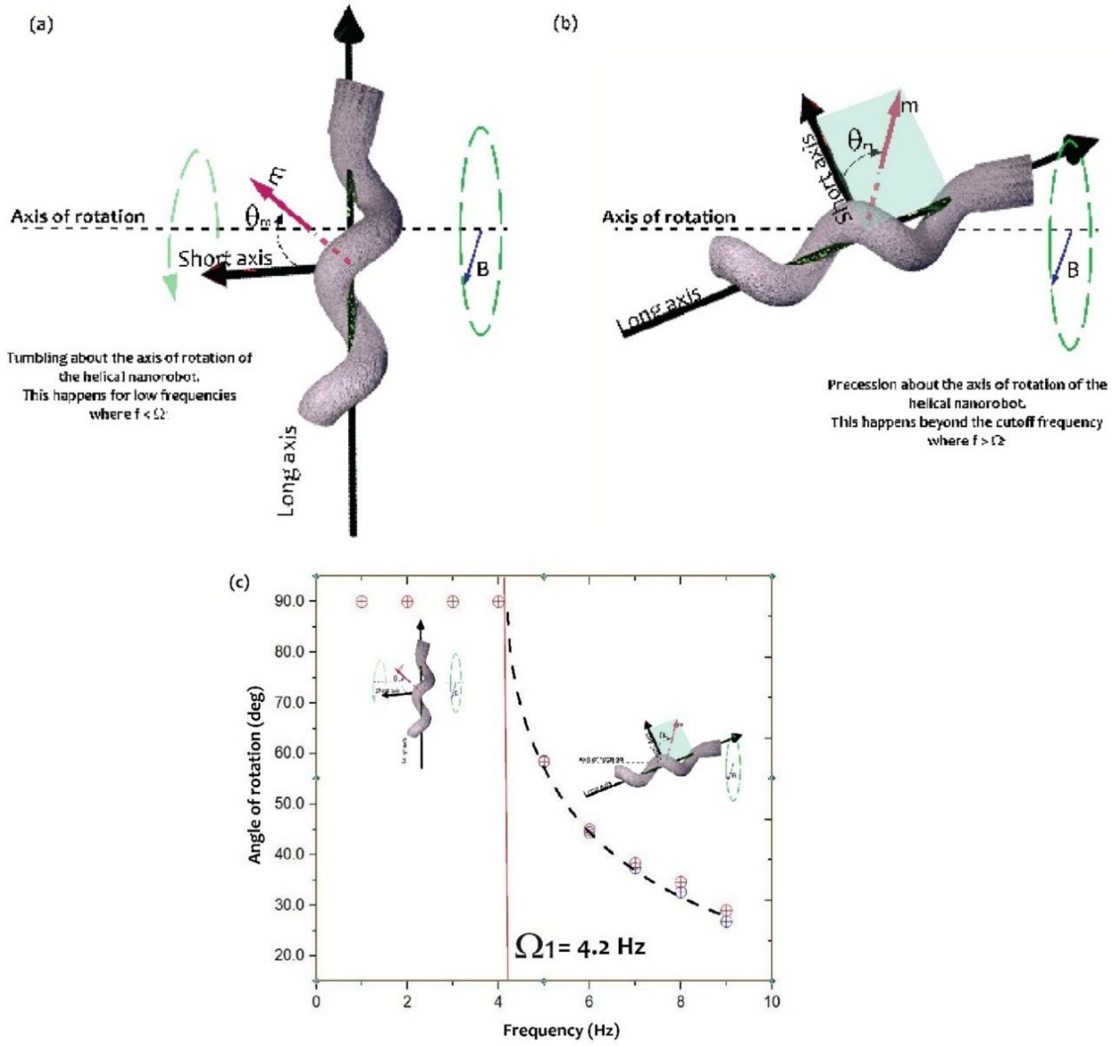

Figure S3: Dynamics of nanomotors. (a) Schematic explaining the tumbling motion of a nanomotor under a rotating magnetic field 'B'. (b) Schematic explaining precessional motion under rotating magnetic field 'B'. (c) Experimental measurements of angle of rotation and the corresponding fit. The data is represented by red and blue crosshair points for two different nanomotors. The cutoff frequency is found to be 4.2Hz

Cell Line	Subtype (ER/PR/HER2)	Reference
HMLE	Immortalized mammary epithelial cell line	Mani, S. A. *et al.*(2008) [4]
MCF-7	Luminal A($ER^{+}/PR^{+/-}/HER2^{-}$)	Sugarman, B. J. *et al.*(1985) [5]
HCC70	Triple Negative ($ER^{-}/PR^{-}/HER2^{-}$)	Gazdar, A. F. *et al.*(1998) [6]
MDA-MB-231	Triple Negative ($ER^{-}/PR^{-}/HER2^{-}$)	Cailleau, R. *et al.*(1974) [7]
S1	Mammary epithelial cell line	Rizki, A. *et al.*(2008) [8]
T4-2	Triple Negative ($ER^{-}/PR^{-}/HER2^{-}$)	Rizki, A. *et al.*(2008) [8]

Table S1

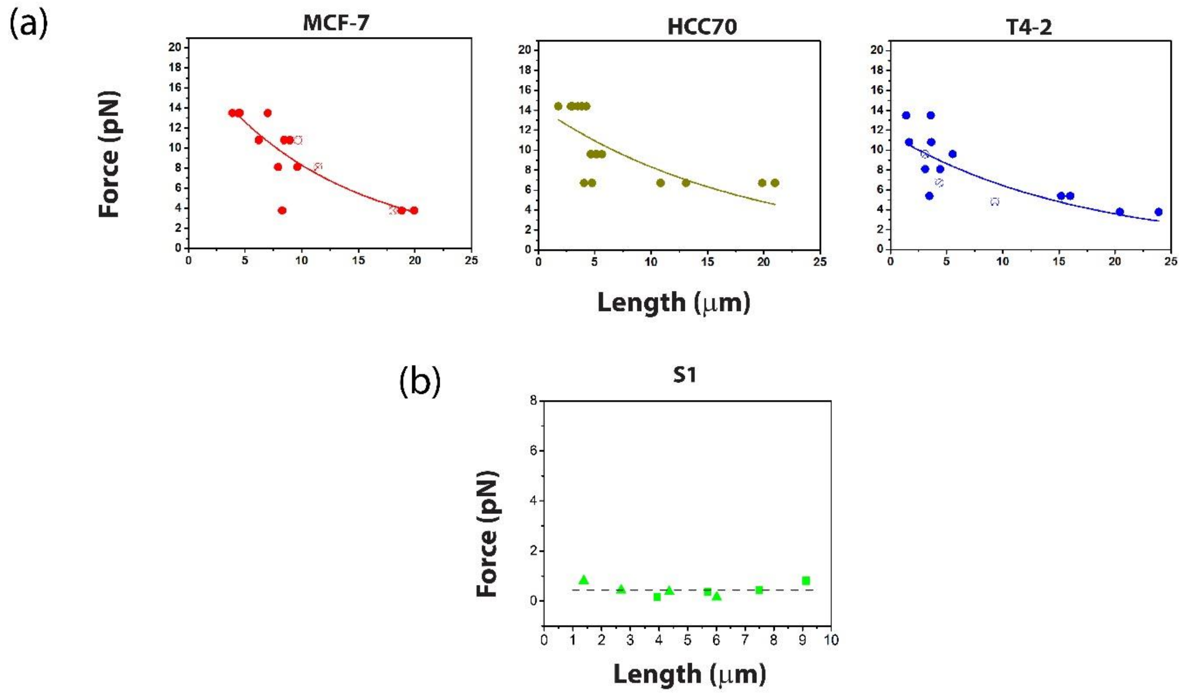

Figure S4: Adhesion force calculation for various cell lines. Plot of average force ($\boldsymbol{F}$) vs adhesion distance ($\boldsymbol{x}$) for (a) three breast cancer cells and (b) a non-transformed cell line is shown. Within each graph, open circles correspond to a single nanomotor, highlighting the self-referenced method of measurement described in the main text.

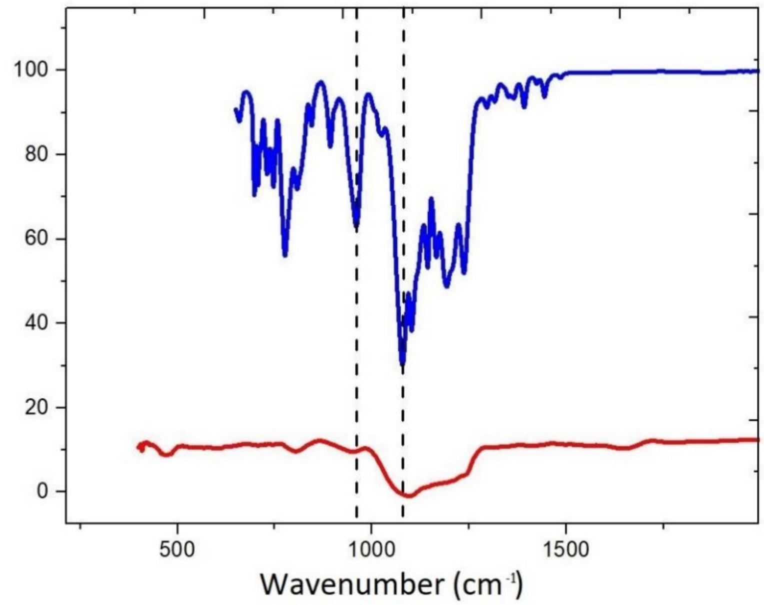

Figure S5: FTIR of PFO coated nanomotors: The blue line represents the transmission mode signal of pure PFO in liquid form. The red line is the reflected signal obtained after subtracting the signal from PFO coated nanomotors from the uncoated nanomotors. The wavelength where PFO absorbs IR coincides with the absorption signal in the coated nanomotors.

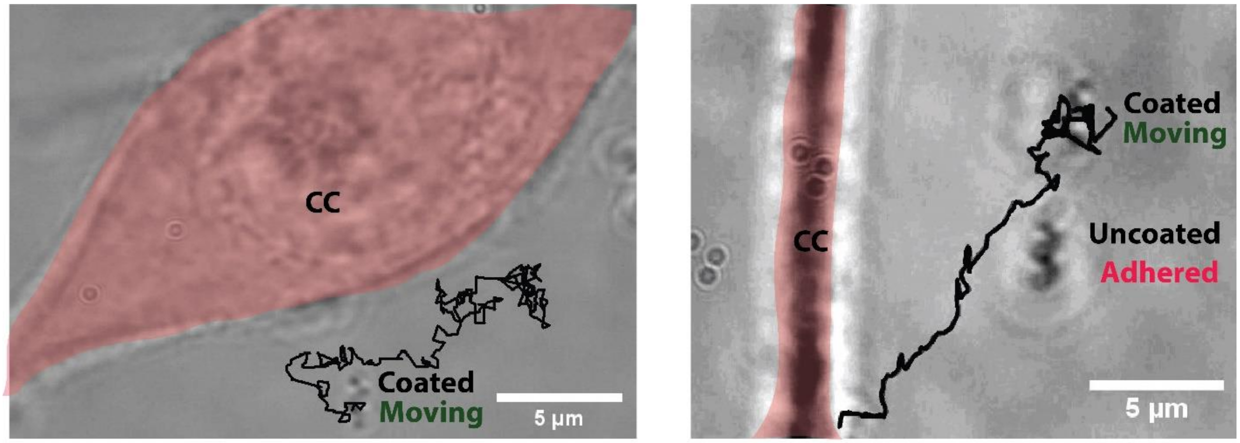

Figure S6: Lack of adhesion in PFO coated nanomotors

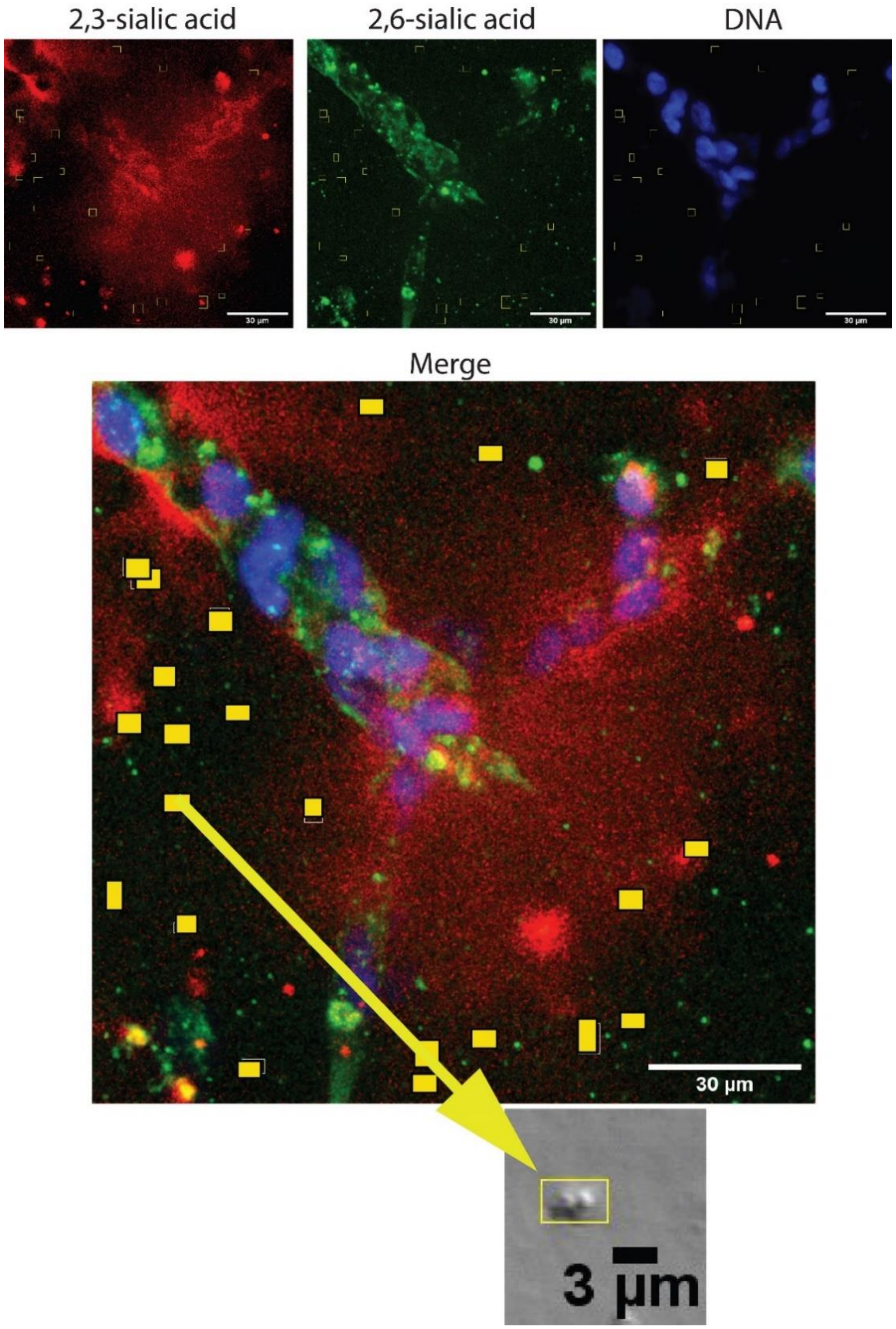

Figure S7: MDA-MB-231(CC) cells showing the coincidence in localization of adhered nanomotors and extracellular α2,3-linked sialic acid (red) in the ECM. (Red: α2,3-linked sialic acid using MAA-TRITC, Green: cell surface staining with α2,6-linked sialic acid using SNA-FITC, Blue: DNA). All nanomotors in various planes have been highlighted in yellow on the maximum intensity z-projection. All scale bars denote 30 µm

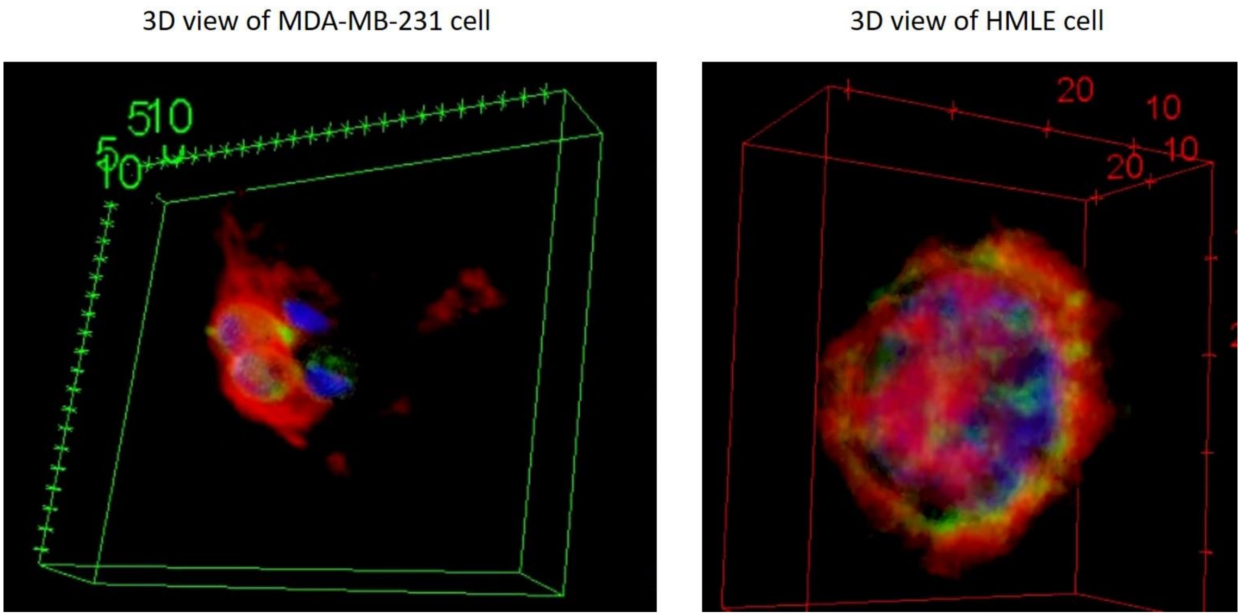

Figure S8: 3D view of MDA-MB-231 and HMLE cell showing the spread of cancer secreted sialic acid. The 3D view is generated from the confocal stacks.

Section S4: Calculation of diffusion of helical nanomotors in rBM .

An elongated object like a cylindrical rod is a good approximation for the geometry of nanomotors. Such elongated bodies will have anisotropy in their diffusivities. We estimated the distance from the point of injection at which nanomotors were found in the rBM to be greater than the diffusive length scale by an order of magnitude. The translational diffusivity of a rod is given by $D_t = \frac{kT(\ln p + C_t)}{3\pi\eta L}$ where k is the Boltzmann constant, T is the ambient temperature, p is the aspect ratio given by $p =$ $\text{(length of the cylinder L)}/\text{(diameter of cylinder d)}$, C_t is a numerical constant given by $C_t = 0.312 + \frac{0.565}{p} - \frac{0.1}{p^2}$, η is viscosity of the fluid[9]. Measuring the effective viscosity of rBM at the length scale of nanomotors is not trivial as length scale dependence of viscosity in a fluid with microscopic heterogeneity is not captured by bulk rheometry[10]. We can calculate to an order, the length a cylinder of length 3 μm and diameter 0.5 μm diffusing in a fluid of viscosity 1Pa.s can travel in 1 hour. With $p = 15,\ C_t = 0.349$

and D_t at room temperature is 4.44×10^{-16}m^2/s. The length traversed by such a particle in 1 hour due to diffusion is $l_p = \sqrt{D_t T}$ =1.2 µm.

References

1. Blaschke, R. J., Howlett, A. R., Desprez, P.-Y., Petersen, O. W. & Bissell, M. J. [25] Cell differentiation by extracellular matrix components. *Methods Enzymol.* **245**, 535–556 (1994).

2. Pal, M. *et al.* Helical nanobots as mechanical probes of intra- and extracellular environments. *J. Phys. Condens. Matter* (2020). doi:10.1088/1361-648X/ab6f89

3. Schindelin, J. *et al.* Fiji: an open-source platform for biological-image analysis. *Nat. Methods* **9**, 676–682 (2012).

4. Mani, S. A. *et al.* The Epithelial-Mesenchymal Transition Generates Cells with Properties of Stem Cells. *Cell* **133**, 704–715 (2008).

5. Sugarman, B. J. *et al.* Recombinant human tumor necrosis factor-α: Effects on proliferation of normal and transformed cells in vitro. *Science (80-.).* **230**, 943–945 (1985).

6. Gazdar, A. F. *et al.* Characterization of paired tumor and non-tumor cell lines established from patients with breast cancer. *Int. J. Cancer* **78**, 766–774 (1998).

7. Cailleau, R., Young, R., Olivé, M. & Reeves, W. J. Breast tumor cell lines from pleural effusions. *J. Natl. Cancer Inst.* **53**, 661–674 (1974).

8. Rizki, A. *et al.* A human breast cell model of preinvasive to invasive transition. *Cancer Res.* **68**, 1378–1387 (2008).

9. Ortega, A. & García de la Torre, J. Hydrodynamic properties of rodlike and disklike particles in dilute solution. *J. Chem. Phys.* **119**, 9914–9919 (2003).

10. Lai, S. K., Wang, Y.-Y., Wirtz, D. & Hanes, J. Micro- and macrorheology of mucus. *Adv. Drug Deliv. Rev.* **61**, 86–100 (2009).
